# Protective Effect of Exogenous Adenosine Triphosphate Against Ocular Toxicity of Linezolid in Rats

**DOI:** 10.3390/life15101587

**Published:** 2025-10-11

**Authors:** Cenap Mahmut Esenulku, Ibrahim Cicek, Ahmet Mehmet Somuncu, Bulent Yavuzer, Esra Tuba Sezgin, Tugba Bal Tastan, Nurinisa Yücel, Ezgi Karatas, Halis Suleyman

**Affiliations:** 1 Department of Ophthalmology, Trabzon Kanuni Education and Research Hospital, Health Sciences Univesity, Trabzon 61250, Turkey; dr.cesenulku@gmail.com (C.M.E.); mehmetsomuncu89@hotmail.com (A.M.S.); 2 Department of Ophthalmology, Faculty of Medicine, Erzincan Binali Yıldırım University, Erzincan 24100, Turkey; ibrahim.cicek@erzincan.edu.tr; 3 Department of Pharmacology, Faculty of Medicine, Erzincan Binali Yıldırım University, Erzincan 24100, Turkey; drbyavuzer@hotmail.com; 4 Program in Anesthesia, Vocational School of Health Services, Erzincan Binali Yıldırım University, Erzincan 24100, Turkey; esra.demir@erzincan.edu.tr; 5 Department of Histology and Embryology, Faculty of Medicine, Erzincan Binali Yıldırım University, Erzincan 24100, Turkey; tbal@erzincan.edu.tr; 6 Pharmacy Services Program, Vocational School of Health Services, Erzincan Binali Yildirim University, Erzincan 24100, Turkey; nurinisa.yucel@erzincan.edu.tr; 7 Department of Ophthalmology, Faculty of Medicine, Agri Ibrahim Cecen University, Agri 04100, Turkey; e.karatas.2015@gmail.com

**Keywords:** adenosine triphosphate, linezolid, ocular toxicity, oxidative stress, rats

## Abstract

Linezolid, a synthetic antimicrobial agent, may induce oxidative damage in ocular tissues, particularly in the optic nerve. Adenosine triphosphate (ATP) is involved in the production of antioxidants that scavenge and neutralize reactive oxygen species. This study aims to evaluate the potential protective effect of exogenous ATP against linezolid-induced ocular damage in rats, in comparison with methylprednisolone. Wistar-type rats were divided into five groups as follows: healthy (HG), ATP-only (ATPG), linezolid-only (LZDG), ATP + linezolid (ATLDG), and methylprednisolone + linezolid groups (MPLDG). Oxidative stress markers, antioxidant biomarkers, and proinflammatory cytokines were analyzed in isolated ocular tissues. Optic nerve tissue was also evaluated histopathologically. Linezolid administration increased the oxidative stress marker MDA and the proinflammatory cytokine TNF-α, while decreasing antioxidant parameters such as tGSH, SOD and CAT in rat ocular tissues, compared to the healthy group. However, it did not significantly alter serum troponin I levels. Histopathological analysis revealed that linezolid induced oxidative damage and inflammation in optic nerve tissue, with marked glial alterations. ATP administration reduced linezolid-induced oxidative stress in ocular tissue, as indicated by decreased MDA levels. It also enhanced antioxidant defenses by increasing tGSH, SOD, and CAT levels. In addition, ATP lowered proinflammatory cytokine levels, thereby alleviating inflammation. These effects collectively contributed to the restoration of biochemical parameters toward normal levels. In addition, ATP mitigated linezolid-induced optic nerve damage and glial alterations. The critical role of ATP in reducing oxidative stress, restoring antioxidant balance, and suppressing inflammation may represent a promising therapeutic approach for linezolid-induced ocular toxicity.

## 1. Introduction

Linezolid is a synthetic oxazolidinone antimicrobial drug approved for the treatment of infections complicated by bacterial pneumonia, Gram-positive microorganisms, vancomycin-resistant enterococci, skin and bacteremia [1]. The drug is considered a useful antibiotic in the intensive care unit against infections caused by the mentioned microorganisms [2]. Linezolid binds to the bacterial 23S ribosomal RNA of the 50S subunit, thus preventing the formation of a functional 70S initiation complex [1,3]. This activity essentially suppresses protein production and hinders the proliferation of bacteria. Linezolid is bacteriostatic towards enterococci and staphylococci, and also bactericidal towards most strains of streptococci [4]. Linezolid is a particularly effective drug for treating drug-resistant infections [5]. It is preferred for the treatment of orthopedic infections due to its broad-spectrum activity and good penetration into bone tissue [6]. However, linezolid can cause serious side effects such as thrombocytopenia, peripheral neuropathy, lactic acidosis and optic neuropathy and as well as potential vision loss [7]. Therefore, physicians recommend immediate discontinuation of treatment to prevent irreversible vision loss in patients on long-term linezolid therapy [8]. The literature does not provide any information connecting the ocular toxicity of linezolid to oxidative stress. However, it was reported that linezolid increased blood malondialdehyde (MDA) levels [9]. In a further study, linezolid was reported to cause oxidative damage in liver and kidney tissue by altering the oxidant antioxidant balance in favor of oxidants [10]. As is well established, MDA is a byproduct of reactive oxygen species (ROS), and increased ROS levels can trigger the transcription of tumor necrosis factor-alpha (TNF-α) along with other proinflammatory cytokines [11]. The precise mechanism of linezolid’s ocular toxicity remains unclear. Nevertheless, the research indicates that linezolid diminishes adenosine triphosphate (ATP) levels in HepG2-C3A cells [12]. As is well known, ATP is a nucleoside triphosphate made up of adenine, a ribose sugar and three phosphate groups [13]. ATP is involved in the production of antioxidants that eliminate and neutralize ROS [14]. Furthermore, ATP is a source of energy for the synthesis of antioxidants [15]. ATP has been reported to prevent MDA overproduction and endogenous glutathione (tGSH) depletion, protect tissues from oxidative damage and normalize their function [16]. Current evidence indicates that the ocular toxicity of linezolid may result from a reduction in both extra- and intracellular ATP reserves. In the literature, studies have been conducted on substances such as pyridoxine and silymarin for the prevention of linezolid-induced oxidative stress [9,10]. However, the protective effects of ATP against linezolid-induced oxidative stress will be comprehensively investigated for the first time, and the potential advantages of ATP will be discussed. Methylprednisolone is a corticosteroid commonly used in the treatment of various eye diseases due to its anti-inflammatory and immunosuppressive properties. It has been suggested that it may be beneficial in reducing oxidative stress and controlling inflammation, especially in cases of optic nerve damage. Experimental studies have shown that methylprednisolone exhibits protective effects by reducing oxidative damage in ocular tissues [17]. Furthermore, there is a lack of experimental or clinical research in the literature investigating the protective effect of ATP against linezolid-induced ocular damage. This study aims to assess the potential protective effect of exogenous ATP against linezolid-induced ocular damage in rats and compare it with methylprednisolone.

## 2. Materials and Methods

### 2.1. Experimental Animals

Thirty male albino Wistar-type rats, weighing 270–285 g and aged 9–10 wks, were utilized for the experiment. Animals were obtained from the Experimental Animal Application and Research Centre (Erzincan Binali Yıldırım University, Erzincan, Turkey). The animals were randomly allocated into five groups (*n* = 6), each exhibiting a comparable average body weight. Animals were randomly assigned to treatment and control groups using simple randomization. The randomization sequence was generated using Microsoft Excel’s RAND function. Each animal was given a random number and groups were formed based on the ascending order of these numbers. Prior to the experiment, the rats were housed in conventional wire cages for laboratory animals (cages; floor area: 1925 cm^2^; height: 20 cm; width: 35 cm; length: 55 cm) in groups of six to acclimatize to the laboratory environment, under a 12 h light/dark cycle, at a temperature of 22 °C and with a humidity level of 30–70%. Animals were provided ad libitum access to tap water and regular pellet feed (Experimental Animal Feed; Bayramoglu Stock Company, Erzurum, Turkey). Six animals were housed per cage in a total of five cages, and to minimize pain and distress, inhalation anesthesia with sevoflurane was administered prior to all invasive procedures. All experiments were performed in compliance with the European Parliament and Council Directive 2010/63/EU (Number of approvals: 2016-24-199) and the ARRIVE directives [18]. All procedures were conducted with the approval of the Local Ethics Committee for Animal Experiments (Erzincan Binali Yıldırım University, Erzincan, Turkey; Meeting Date: 26 December 2024; Meeting No: 2024/12; Decision No: 57).

### 2.2. Chemicals

The substances utilized in the experiment included sodium thiopental (Pental Sodyum^®^ 0.5 g vial) acquired from IE Ulagay Pharmaceutical Industry (Istanbul, Turkey); ATP (ATP^®^ 10 mg/mL 1 mL vial) sourced from Zdorovye Narodu Pharmaceutical Ltd., Co. (Kharkiv, Ukraine); methylprednisolone (Prednol-L^®^ 40 mg lyophilized injectable ampoule) sourced from Mustafa Nevzat Pharmaceutical Industry (Istanbul, Turkey); and linezolid (Zyvoxid^®^ 600 mg tablet) obtained from Pfizer Pharmaceutical Co. (Istanbul, Turkey).

### 2.3. Experimental Groups

The sample size was determined to utilize the smallest number of animals permissible in accordance with the 3R (Reduction, Refinement and Replacement) requirements [19]. The rats were randomly assigned to five experimental groups: the healthy control group (HG), the ATP-only group (ATPG; 4 mg/kg, intraperitoneally), the linezolid-only group (LZDG; 125 mg/kg, orally), the combination group receiving both ATP (4 mg/kg, intraperitoneally) and linezolid (125 mg/kg, orally) (ATLDG) and the methylprednisolone (30 mg/kg, intraperitoneally) and linezolid (125 mg/kg, orally) group (MPLDG).

### 2.4. Experimental Procedure

ATP was administered intraperitoneally (4 mg/kg) to the ATPG (*n* = 6) and ATLDG (*n* = 6) groups once daily for two weeks. Previous evidence suggests that ATP at this dosage may confer protection against oxidative damage in optic nerve tissue [20]. Methylprednisolone was administered intraperitoneally at a dose of 30 mg/kg to the MPLDG group (*n* = 6). On subsequent days, the treatment was continued with gradually reduced doses over a two-week period [17]. The LZDG (*n* = 6) group and the HG (*n* = 6) group were given saline (0.9% NaCl) as a solvent in the same manner. One h following the administration of ATP, methylprednisolone, and the solvent, linezolid was orally by gavage delivered at a dosage of 125 mg/kg to the LZDG, ATLDG and MPLDG groups. This linezolid administration protocol was repeated twice daily at 12 h intervals for a duration of two weeks. Oxidative stress has been reported to occur at this dosage of linezolid in prior investigations [21]. Given that linezolid is commonly used twice per day over a 14-day course in clinical settings, the present study focused on investigating the toxicity emerging during this timeframe [7]. At the conclusion of this period, the animals were euthanized using a high-dose anesthetic (50 mg/kg thiopental sodium), and their eyes were excised. Biochemical indices of oxidants (MDA), antioxidants (tGSH, superoxide dismutase (SOD), catalase (CAT)) and proinflammatory cytokine (TNF-α) were assessed in the excised ocular tissues. Troponin I (TP-I) levels were determined in blood samples collected from the tail veins of the animals. The optic nerve tissues were subjected to histological examination. The experimental results of all groups of animals were evaluated by means of intergroup comparisons.

### 2.5. Biochemical Analysis

#### 2.5.1. Preparation of Samples

Approximately 70 milligrams of ocular tissue was excised from each rat and thoroughly rinsed with 0.9% NaCl solution. The tissues were then homogenized using a high-speed homogenizer under ice-cold conditions. Following homogenization, 2 mL of 1.15% potassium chloride (KCl) buffer solution (pH 7.4) was added to each sample. The homogenates were subsequently centrifuged at 10,000× *g* for 15 min at 4 °C. The resulting supernatants were collected and used for the determination of MDA and tGSH levels, as well as for the measurement of SOD and CAT enzyme activities. All biochemical measurements from tissue samples were expressed as milligrams per gram of total protein to ensure standardization across groups.

#### 2.5.2. Determination of MDA, GSH Levels, SOD and CAT Activities in Ocular Tissue

Rat ELISA (enzyme-linked immunosorbent assay) kits for quantifying MDA, GSH, and SOD parameters (MDA product number: 10009055; tGSH product number: 703002; SOD product number: 706002; Cayman Chemical Co., Ann Arbor, MI, USA) were acquired. Each analysis was performed in line with the directives of the particular kit. The CAT determination was conducted following the methodology suggested by Goth [22]. Protein concentrations were determined using the Bradford method, which relies on the binding of Coomassie Brilliant Blue G-250 dye to proteins. The resulting complex was quantified spectrophotometrically by measuring absorbance at 595 nm [23].

#### 2.5.3. Ocular Tissue TNF-α Determination

Tissue samples were first weighed, then sectioned and immediately frozen in liquid nitrogen. The frozen tissues were homogenized using a mortar and pestle. After thawing, the homogenates were kept at 2–8 °C. Phosphate-buffered saline (PBS, pH 7.4) was added at a ratio of 1:10 (*w*/*v*), followed by vortexing for 10 s. The mixture was then centrifuged at 10,000× *g* for 20 min, and the supernatant was carefully collected. Tissue TNF-α concentrations (ng/L) were quantified using a commercially available ELISA kit (Eastbiopharm Co., Ltd., Hangzhou, China), in accordance with the manufacturer’s instructions.

#### 2.5.4. Blood Serum TP-I Determination

Blood serum samples obtained from the animals were analyzed for TP-I levels using the VIDAS^®^ TP-I Ultra kit (Biomérieux, Craponne, Lyon, France) based on the Enzyme-Linked Fluorescent Assay (ELFA) principle. All assay procedures were carried out automatically by the VIDAS^®^ system (Biomérieux, Craponne, Lyon, France) using the pre-packaged test reagents provided with the kit, in accordance with the manufacturer’s protocol.

### 2.6. Histopathological Method

All optic nerve tissue samples were initially fixed in a 10% formaldehyde solution for evaluation via light microscopy. Following the identification process, the tissue samples were subjected to a 24 h wash in tap water within cassettes. The samples were subsequently treated with conventional alcohol grades (70%, 80%, 90%, and 100%) to eliminate water from the tissues. The tissues were subsequently processed with xylol and embedded in paraffin. Sections measuring four to five microns were obtained from the paraffin blocks, followed by the application of a hematoxylin–eosin staining procedure. The photographs were captured following assessment by the Olympus DP2-SAL firmware program (Olympus^®^ Inc., Tokyo, Japan). The histopathological alterations in optic nerve tissue included destruction, an increase in astrocyte cell population, edema or vacuolization, and vascular dilation or congestion. Serial sections were obtained from tissue samples collected from six animals in each group (*n* = 6). In each section, one central and five peripheral regions were selected and analyzed at 400× magnification. Accordingly, a total of 36 images were evaluated per group. Damage in the samples was graded according to the following criteria: 0-none, 1-mild, 2-moderate and 3-severe [19,24,25]. A pathologist, blinded to the study groups, conducted the histopathological evaluation.

### 2.7. Statistical Analysis

All biochemical statistical analyses were conducted utilizing the IBM SPSS Statistical Program for Windows (IBM Corp., V27.0, 2020 release, Armonk, NY, USA). Figures were produced with the GraphPad Prism software (GraphPad Software, V8.0.1, 2018 edition, San Diego, CA, USA). All biochemical data are stated as mean ± standard deviation. The normality assumption was assessed using the Shapiro–Wilk test, while the homogeneity of variances assumption examined by Levene’s test. The one-way ANOVA or Welch’s ANOVA test was employed to assess the mean differences among the groups. Pairwise comparisons of these tests were conducted using Tukey’ s honestly significant difference (HSD) or Games Howell test. All histopathological results are reported as median, minimum, and maximum values. The disparity between the groups regarding median values in histopathologic assessment was ascertained utilizing the Kruskal–Wallis test, a nonparametric method. Dunn’s test with Bonferroni adjustment was employed for pairwise group comparisons, and the adjusted *p*-values are provided. Statistical significance was defined as a probability value of *p* < 0.05.

## 3. Results

### 3.1. Biochemical Results

#### 3.1.1. MDA and tGSH Level Analysis Results

**Figure 1:**
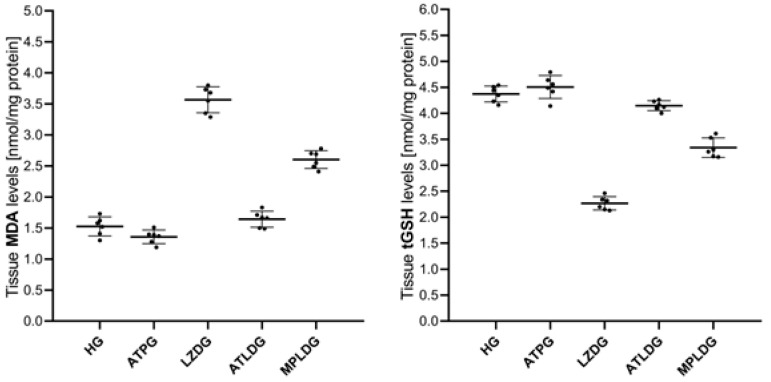
Effects of adenosine triphosphate, methylprednisolone, and linezolid on MDA and tGSH levels in rat ocular tissues. Data are expressed as mean ± SD (standard deviation). Statistical analyses were performed using the one-way ANOVA test followed by Tukey’s honestly significant difference (HSD) test as post hoc. *n* = 6 for each group. ATP: adenosine triphosphate; HG: healthy group; ATPG: ATP-only group; LZDG: linezolid-only group; ATLDG: ATP + linezolid group; MPLDG: methylprednisolone + linezolid group; MDA: malondialdehyde; tGSH: total glutathione.

Figure 1 and Table 1 indicate a small reduction in MDA levels within the eye tissues of the ATPG. The disparity in MDA levels between HG and ATPG was deemed minor (*p* = 0.328). A significant rise in MDA levels was seen in the eye tissue of the LZDG group compared to HG and ATPG (respectively, *p* < 0.001; *p* < 0.001). However, ATP (ATLDG, *p* < 0.0001) more effectively suppressed the linezolid-induced increase in ocular MDA levels compared to methylprednisolone (MPLDG, *p* < 0.001).

Despite the tGSH level in the eye tissue of ATPG being higher than that of HG, the difference was deemed statistically insignificant (*p* = 0.613). The cohort exhibiting the lowest tGSH level was LZDG, but ATP significantly prevented the linezolid-induced depletion of tGSH compared to methylprednisolone (ATLDG, *p* < 0.0001 vs. MPLDG, *p* < 0.001; Figure 1; Table 1).

#### 3.1.2. SOD and CAT Analysis Results

**Figure 2:**
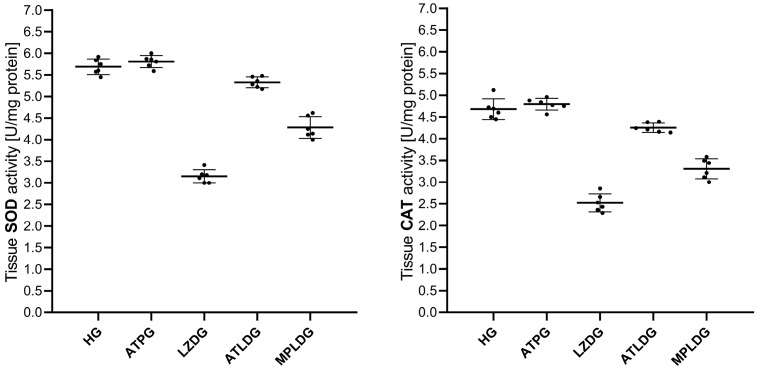
Effects of adenosine triphosphate, methylprednisolone, and linezolid on SOD and CAT activities in rat ocular tissues. Data are expressed as mean ± SD (standard deviation). Statistical analyses were performed using the one-way ANOVA test followed by Tukey’s honestly significant difference (HSD) test as post hoc. *n* = 6 for each group. ATP: adenosine triphosphate; HG: healthy group; ATPG: ATP-only group; LZDG: linezolid-only group; ATLDG: ATP + linezolid group; MPLDG: methylprednisolone + linezolid group; SOD: superoxide dismutase; CAT: catalase.

The activities of SOD (*p* = 0.762) and CAT (*p* = 0.843) were elevated in the eye tissues of ATPG; however, this elevation was statistically insignificant in comparison to HG. The activities of SOD (respectively, *p* < 0.001; *p* < 0.001) and CAT (respectively, *p* < 0.001; *p* < 0.001) in the eye tissues of LZDG were markedly diminished compared to HG and ATPG. In ATLDG and MPLDG, the reduction in SOD (*p* < 0.0001; *p* < 0.001, respectively) and CAT (*p* < 0.0001; *p* < 0.001, respectively) was markedly suppressed (Figure 2; Table 1).

#### 3.1.3. Ocular Tissue TNF-α Assay Results

**Figure 3:**
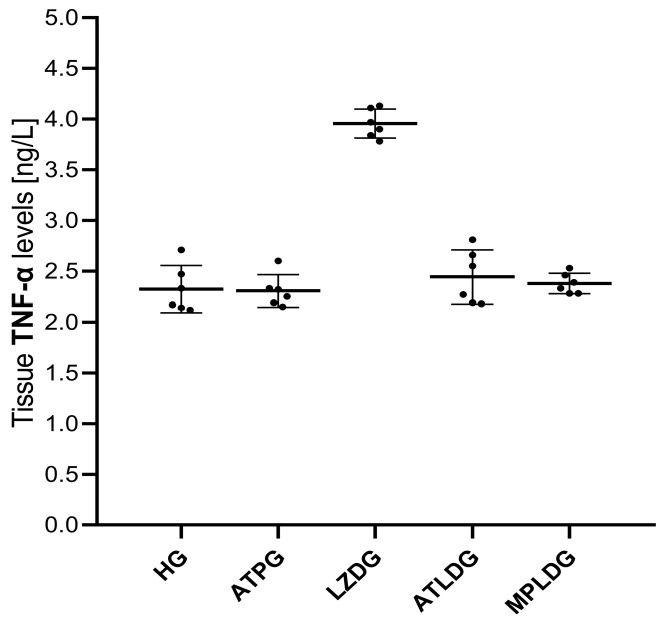
Effects of adenosine triphosphate, methylprednisolone, and linezolid on TNF-α levels in rat ocular tissues. Data are expressed as mean ± SD (standard deviation). Statistical analyses were performed using the one-way ANOVA test followed by Tukey’s honestly significant difference (HSD) test as post hoc. *n* = 6 for each group. ATP: adenosine triphosphate; HG: healthy group; ATPG: ATP-only group; LZDG: linezolid-only group; ATLDG: ATP + linezolid group; MPLDG: methylprednisolone + linezolid group; TNF-α: tumor necrosis factor-alpha.

As presented in Figure 3 and Table 1, linezolid administration led to a significant increase in TNF-α levels in the optic nerve tissue compared to both the HG (*p* < 0.001) and ATPG (*p* < 0.001) groups. While ATP administration did not result in a significant change in TNF-α levels in the ocular tissue of the HG group (*p* = 1.000), it significantly suppressed the elevation observed in the LZDG group (*p* < 0.001). Methylprednisolone (MPLDG, *p* < 0.001) significantly attenuated the linezolid-induced increase in TNF-α levels.

#### 3.1.4. Evaluation of TP-I Levels in Blood Serum

**Figure 4:**
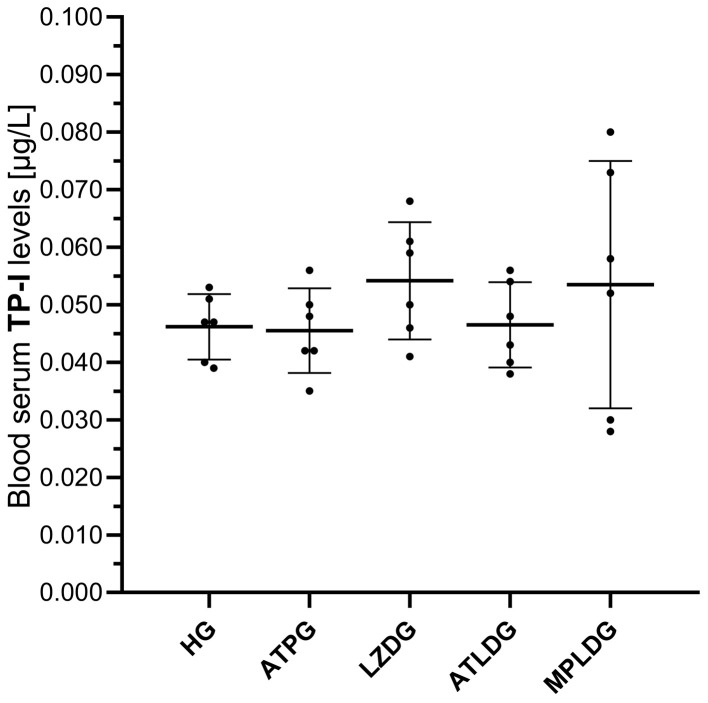
Effects of adenosine triphosphate, methylprednisolone, and linezolid on TP-I levels in rat blood serum. Data are expressed as mean ± SD (standard deviation). Statistical analyses were performed using the Welch ANOVA test followed by Games-Howell test as post hoc. *n* = 6 for each group. ATP: adenosine triphosphate; HG: healthy group; ATPG: ATP-only group; LZDG: linezolid-only group; ATLDG: ATP + linezolid group; MPLDG: methylprednisolone + linezolid group; TP-I: troponin I.

Linezolid administration did not significantly affect TP-I levels in rat blood serum. No statistically significant differences in TP-I concentrations were observed among the experimental groups (Figure 4 and Table 1).

### 3.2. Histopathological Results

**Figure 5:**
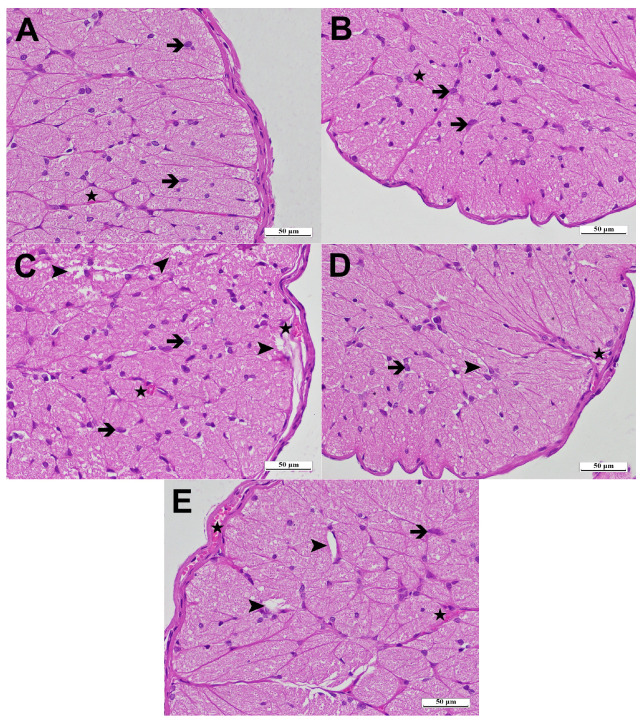
Hematoxylin–eosin staining of the optic nerve tissue (**A**) In the HG group (×400). ➜: astrocyte; ★: blood capillary. (**B**) In the ATPG group (×400). ➜: astrocyte; ★: blood capillary. (**C**) In the LZDG group (×400). ➜: hypertrophied and degenerated astrocyte; ★: blood capillary; ➤: severe edema and vacuolization. (**D**) In the ATLDG group (×400). ➜: astrocyte; ★: blood capillary; ➤: mild edema and vacuolization. (**E**) In the MPLDG group (×400). ➜: hypertrophied and degenerated astrocyte, ★: blood capillary, ➤: moderate edema and vacuolization. ATP: adenosine triphosphate; HG: healthy group; ATPG: ATP-only group; LZDG: linezolid-only group; ATLDG: ATP + linezolid group; MPLDG: methylprednisolone + linezolid group.

Upon examining optic nerve tissue sections from HG using a light microscope, no pathological abnormalities were seen in the connective tissue surrounding the optic nerve, the blood vessels within the nerve trabeculae, or the extensions of astrocytes. Axons exhibited eosinophilia, while glial cells demonstrated basophilia (Figure 5A). Upon analysis of the optical nerve tissue sections from ATPG, no pathological alterations were seen in the connective tissue surrounding the optical nerve, the nerve trabeculae, or the blood vessels in these regions. The astrocytes, identified in typical nerve tissue structures, exhibited basophilic staining, whereas their extensions had eosinophilic staining (Figure 5B). In the optic nerve samples from LZDG, extensive vacuolation and edema were noted. Despite the usual thickness of the surrounding connective tissue, notable dilatation and congestion were observed in the blood vessels inside the connective tissue and nerve trabeculae. Hypertrophic and degraded astrocytes were notably enhanced in the tissue (Figure 5C). In the optical nerve tissue sections of the ATLDG group, the adjacent connective tissue, astrocytes, and blood vessels exhibited normalcy, akin to the healthy group, whereas the vacuolization and edema noted in the severe damage group were found to be mild (Figure 5D). In optic nerve tissue samples from the MPLDG group treated with methylprednisolone, moderate vacuolization and edema, vascular dilatation with congestion, and astrocyte degeneration were evident (Figure 5E). The histopathological grading data derived from rat optic nerve tissue is presented in Table 2.

## 4. Discussion

This study examined the biochemical and histopathological effects of linezolid-induced oxidative stress on ocular toxicity. In addition, the protective effect of ATP against linezolid-induced ocular toxicity was investigated, and evaluated in comparison with methylprednisolone. Our findings indicated that linezolid treatment induced substantial biochemical and histopathological alterations in ocular tissue; however, ATP delivery markedly mitigated these changes. Despite extensive documentation of linezolid’s ocular toxicity in the literature, the underlying mechanism of this adverse impact remains inadequately clarified. Certain case studies in the current literature offer significant insights into the scope and mechanisms of linezolid-induced effects. Yun et al. (2025) reported that in cases of optic nerve damage following prolonged linezolid therapy, discontinuation of the treatment resulted in a significant improvement in vision; however, this improvement was not observed in all patients [26]. Ishii et al. (2016) [27] documented a case in which linezolid induced uncommon pathological alterations and identified damage to the inner retinal layers. This discovery indicates that the deleterious effects of linezolid extend beyond the optic nerve, potentially affecting various retinal components as well [27]. Linezolid-associated optic neuropathy typically arises after prolonged use (>28 days), and early cessation of treatment positively influences the prognosis [28]. Han et al. (2023) documented an uncommon example of concurrent toxic optic neuropathy and cataract formation resulting from linezolid administration [29]. The data indicate that linezolid may lead to a broad range of ocular problems. Nonetheless, preclinical investigations regarding the ocular toxicity of linezolid yield fewer comprehensive data in comparison to case reports [9,30].

The main cause of linezolid-induced ocular toxicity, especially optic neuropathy, is thought to be drug-induced mitochondrial dysfunction [31]. Linezolid, which acts by inhibiting bacterial ribosomal protein synthesis, has been scientifically proven to disrupt mitochondrial activity in human cells [32]. In the optic neurons, a significant amount of energy is generated by mitochondria via oxidative phosphorylation, necessitating the transfer of electrons along a complex chain [33]. When electron transport is unsuccessful, ROS are generated [34]. The combination of energy deprivation and oxidative stress causes cytochrome C to leak out of mitochondrial pores, triggering nerve damage [35]. The main role of oxidative stress in the development of ocular pathologies is associated with lipid peroxidation (LPO) [36]. MDA, which is produced during LPO, is a highly reactive oxidant molecule and is used as an important biochemical marker to determine long-term cell damage and oxidative stress [37]. In our study, it was found that oxidant MDA levels in the eye tissue were significantly increased in the linezolid-treated group. In a recent study by Kendir et al. (2023) [9], it was shown that MDA levels were significantly increased in rat erythrocytes after linezolid administration. This study also states that including the antioxidant molecule pyridoxine into the treatment reduces the oxidative stress brought on by linezolid, hence preventing hematological and hepatological damage [9]. The literature data align with our study’s findings and indicate that linezolid may induce harmful effects in ocular tissue by causing oxidative imbalance.

Living organisms have evolved a comprehensive endogenous antioxidant defense system to prevent the formation of ROS or limit their harmful effects [38]. Müller (glial) cells in the retina contain high amounts of GSH, which may exert protective effects on retinal neurons under oxidative stress conditions [39]. In the literature, there are studies suggesting that GSH assumes an eminent role in the scavenging of ROS in ocular tissues [40,41]. In this study, we measured tGSH levels, which function as an indicator of endogenous antioxidant defense in optic nerve tissue. Our experimental findings, consistent with existing literature, indicate a substantial reduction in tGSH levels in the ocular tissue of the group treated with linezolid. Moreover, concomitant with the reduction in tGSH levels, a significant decline in the activity of the ROS-scavenging antioxidant enzymes (SOD and CAT) was noted in the ocular tissue of the group treated with linezolid. A study by Wang et al. found that linezolid induced a dose-dependent reduction in the activity of SOD and CAT enzymes in rat serum. In the same study, the addition of an antioxidant molecule to the treatment was shown to prevent hematologic toxicity by reducing the oxidative stress induced by linezolid [21]. The literature findings align with our study’s data and indicate that linezolid exacerbates oxidative imbalance by inhibiting antioxidant defense mechanisms.

In the present study, TNF-α levels were evaluated in optic nerve tissue to assess the proinflammatory response associated with linezolid exposure. Our findings revealed a significant increase in TNF-α concentration in the optic nerve of rats treated with linezolid, which appeared to parallel the elevation in MDA levels, a well-established marker of lipid peroxidation and oxidative stress. These results suggest that linezolid may induce inflammatory responses in ocular tissues, likely mediated through oxidative stress mechanisms. Interestingly, serum TP-I levels remained unchanged across all groups, indicating that the inflammatory changes were localized rather than systemic in nature. This localized effect aligns with the known mechanism whereby MDA facilitates the activation of nuclear factor kappa B (NF-κB), subsequently promoting the transcription of proinflammatory cytokines such as TNF-α [11]. Notably, ATP administration did not significantly alter TNF-α level in the optic nerve tissue of healthy animals; however, it effectively attenuated the linezolid-induced elevation of TNF-α. These findings are in agreement with earlier studies reporting that ATP exhibits protective effects on ocular tissues by suppressing oxidative stress and proinflammatory cytokine expression, thereby mitigating retinal inflammation and damage [42].

Due to their very large size relative to other human nerve cells and the high frequency of visual input transmission, eye tissue cells require substantial energy. This situation necessitates effective ATP energy production [43,44]. Moreover, ATP is known to be involved not only in energy production but also in critical roles such as scavenging ROS and providing energy for antioxidant synthesis [45]. Furthermore, extracellular ATP mediates its effects primarily through purinergic receptors, which are pivotal in regulating oxidative stress responses and modulating inflammation within ocular tissues [46]. Previous studies have shown that linezolid inhibits mitochondrial ATP synthesis, leading to oxidative stress and cellular energy imbalance [8,12,34,35]. In present study, it was noted that ATP administration suppressed linezolid-induced MDA elevation and increased antioxidant parameters (SOD, CAT, and tGSH) to near normal limits. This finding strongly supports the protective effects of ATP against oxidative stress. In particular, it has been previously reported in the literature that ATP may protect tissues against oxidative damage by promoting glutathione synthesis [16]. Our findings indicate that this approach may also effectively mitigate linezolid-induced toxicity and imply that ATP could serve as a potential therapeutic agent.

In our study, the protective effect of ATP against linezolid-induced ocular toxicity was also evaluated in comparison with methylprednisolone. As is well known, methylprednisolone is a corticosteroid with anti-inflammatory and immunosuppressive properties, commonly used in the treatment of allergic reactions, arthritis, asthma, and multiple sclerosis [47]. Experimental studies have suggested that methylprednisolone may be beneficial in the treatment of optic neuropathy [20]. Our findings revealed that methylprednisolone exerted an antioxidant effect in the ocular tissue. However, its ability to counteract the oxidative imbalance induced by linezolid—namely, an increase in oxidants and a decrease in antioxidants—was less pronounced than that of ATP. Although previous studies have indicated that methylprednisolone may enhance antioxidant activity in ocular tissues [41]. Some clinical reports suggest that intravenous methylprednisolone and oral corticosteroid therapy do not improve linezolid-induced visual impairment [48].

In the present study, methylprednisolone significantly inhibited the linezolid-induced elevation of TNF-α level in the ocular tissue. The absence of a significant difference in TNF-α level between the methylprednisolone and ATP groups suggests that the anti-inflammatory activity of methylprednisolone may be stronger than its antioxidant capacity. This observation is consistent with literature reports indicating that methylprednisolone exerts its anti-inflammatory effects by suppressing the production of proinflammatory cytokines, thereby contributing to the attenuation of optic neuropathy [49].

In our study, the histopathological findings obtained from the optic nerve tissue showed that linezolid caused significant tissue damage in accordance with the biochemical data. Histopathological changes, such as vacuolization, edema, dilation and congestion of blood vessels, were observed in the linezolid group than in the healthy control group and the ATP-only group. These findings indicate that linezolid causes significant structural damage to the optic nerve tissue. Moreover, the prominence of hypertrophic and degenerated astrocytes suggests that linezolid may enhance the glial response. These results are compatible with earlier literature highlighting the adverse effects of long-term use of linezolid on the optic nerve [50,51]. Among the literature, investigations regarding the toxic effects of linezolid affecting the optic nerve are predominantly confined to case reports involving human subjects [26,27,28,29]. Research with animal models is also limited being the most significant work in this field undertaken by Duke et al. involving rabbits. In this study, no significant histopathologic changes were reported in the ocular tissue of linezolid-treated rabbits compared to the saline-treated group [30]. Meanwhile, our study’s data provide an original contribution to the current literature by confirming linezolid-induced optic nerve injury in a rat model through histopathological findings. The minor vacuolization and edema observed in the ATP-treated group, along with the maintenance of normal histological structure in connective tissue and blood vessels, suggest the neuroprotective potential of ATP. These findings indicate that ATP may have a protective function in nerve tissue, aligning with previous studies [52,53]. Histopathological examination of the methylprednisolone group revealed grade-2 vacuolated and edematous areas, vascular dilatation with congestion, and astrocytic degeneration. The less pronounced protective effect of methylprednisolone on tissue morphology, when compared to ATP, may be due to its lower antioxidant potency. This study has certain limitations that should be considered. First, investigating the effect of linezolid and ATP on oxidant-antioxidant markers in optic nerve tissue, together with their association with proinflammatory and anti-inflammatory parameters, could be beneficial for pathogenesis-oriented therapy. Additionally, measuring tissue ATP levels in linezolid-treated animals may help clarify whether linezolid reduces endogenous ATP concentrations. Also, the absence of immunohistochemically analyses in the present study may have restricted the depth of pathological characterization. Additionally, exogenous ATP is inherently unstable and subject to rapid degradation by enzymes such as CD39 and CD73, which limits its bioavailability. ATP is recognized and metabolized by cells through purinergic signaling pathways, also. To overcome these challenges, potential strategies including localized delivery methods, enzyme inhibitors, and nanoparticle-based carrier systems may be developed. Furthermore, the current study did not investigate whether ATP’s antioxidant effects occur directly or via modulation of endogenous antioxidant pathways, representing a mechanistic limitation that warrants further molecular-level investigation. Although no adverse effects were observed in our animal model, exogenous ATP may cause systemic effects such as inflammation, hypotension, or immune activation, depending on the dose and administration route. These risks should be considered in future in vivo studies. Even though there are anatomical differences, the Wistar rat model exhibits comparable susceptibility of the optic nerve to mitochondrial dysfunction and ATP depletion observed in humans, thereby representing a suitable and translational model for investigating linezolid-induced ocular toxicity. Finally, exploring the dose–response relationship of ATP could assist in identifying an optimal therapeutic dose for the prevention of linezolid-induced ocular damage. Further studies addressing these gaps are warranted to better elucidate the underlying mechanisms and therapeutic potential.

## 5. Conclusions

These findings suggest that linezolid may cause oxidative damage in optic nerve tissue and morphologic changes in glial cells. Furthermore, ATP administration demonstrated a stronger potential than methylprednisolone in mitigating the possible neurotoxic effects of linezolid. The critical role of ATP in reducing oxidative stress and regulating antioxidant balance may offer a novel and promising therapeutic approach for linezolid-induced toxicity. Our experimental findings suggest that ATP may be beneficial in the treatment of linezolid-associated optic neuropathy. Moreover, ATP could be considered a preferable alternative to methylprednisolone in managing optic nerve toxicity, which often limits linezolid dosage or necessitates treatment discontinuation in clinical practice.

## Figures and Tables

**Table 1:** Effects of linezolid, adenosine triphosphate, and methylprednisolone on oxidative stress markers and proinflammatory cytokines in rat ocular tissue, and on cardiac biomarkers in serum.

	Biochemical Variables					
Groups	MDA * (nmol/mg Protein)	tGSH * (nmol/mg Protein)	SOD * (U/mg Protein)	CAT * (U/mg Protein)	TNF-α * (ng/L)	TP-I ** (µg/L)
HG	1.53 ± 0.15	4.37 ± 0.15	5.69 ± 0.18	4.68 ± 0.24	2.32 ± 0.23	0.046 ± 0.006
ATPG	1.36 ± 0.11	4.51 ± 0.22	5.81 ± 0.14	4.79 ± 0.14	2.31 ± 0.16	0.046 ± 0.007
LZDG	3.57 ± 0.21	2.27 ± 0.13	3.15 ± 0.15	2.52 ± 0.21	3.96 ± 0.14	0.054 ± 0.010
ATLDG	1.64 ± 0.13	4.15 ± 0.09	5.33 ± 0.13	4.25 ± 0.11	2.44 ± 0.27	0.047 ± 0.007
MPLDG	2.60 ± 0.14	3.34 ± 0.19	4.28 ± 0.25	3.31 ± 0.23	2.38 ± 0.10	0.054 ± 0.021
Group comparisons	*p*-values					
HG vs. ATPG	0.328	0.613	0.762	0.843	1.000	1.000
HG vs. LZDG	<0.001	<0.001	<0.001	<0.001	<0.001	0.494
HG vs. ATLDG	0.679	0.152	0.013	0.006	0.809	1.000
HG vs. MPLDG	<0.001	<0.001	<0.001	<0.001	0.987	0.918
ATPG vs. LZDG	<0.001	<0.001	<0.001	<0.001	<0.001	0.484
ATPG vs. ATLDG	0.025	0.006	<0.001	<0.001	0.727	0.999
ATPG vs. MPLDG	<0.001	<0.001	<0.001	<0.001	0.965	0.901
LZDG vs. ATLDG	<0.0001	<0.0001	<0.0001	<0.0001	<0.001	0.592
LZDG vs. MPLDG	<0.001	<0.001	<0.001	<0.001	<0.001	1.000
ATLDG vs. MPLDG	<0.001	<0.001	<0.001	<0.001	0.975	0.935
F value	224.853	196.137	245.195	153.945	84.290	0.836 ^a^
df (df1/df2)	4/25	4/25	4/25	4/25	4/25	4/12.216
*p*	<0.001	<0.001	<0.001	<0.001	<0.001	0.527

Results are expressed as mean ± standard deviation. * Statistical analyses were performed using one-way ANOVA followed by Tukey’s honestly significant difference (HSD) test for post hoc comparisons, as the assumption of homogeneity of variances was met. ** Statistical analyses were performed using Welch’s ANOVA followed by the Games-Howell test for post hoc comparisons, due to the violation of the homogeneity of variances assumption. ^a^ Asymptotically F distributed (Welch ANOVA). For all groups *n* = 6. ATP: adenosine triphosphate; HG: healthy group; ATPG: ATP-only group; LZDG: linezolid-only group; ATLDG: ATP + linezolid group; MPLDG: methylprednisolone + linezolid group; MDA: malondialdehyde; tGSH: total glutathione; SOD: superoxide dismutase; CAT: catalase; TNF-α: tumor necrosis factor-alpha; TP-I: troponin I; df: degree of freedom.

**Table 2:** Evaluation of histopathological grading data from rat optic nerve tissue.

Groups	Histopathological Grading Data			
	Destruction	Increase in Astrocyte Cell Population	Edema/Vacuolization	Vascular Dilatation/Congestion
HG	0.00 (0.00–0.00)	0.00 (0.00–0.00)	0.00 (0.00–0.00)	0.00 (0.00–0.00)
ATPG	0.00 (0.00–0.00)	0.00 (0.00–0.00)	0.00 (0.00–0.00)	0.00 (0.00–0.00)
LZDG	3.00 (2.00–3.00)	2.00 (1.00–3.00)	2.00 (1.00–3.00)	2.50 (2.00–3.00)
ATLDG	0.00 (0.00–1.00)	0.00 (0.00–1.00)	0.00 (0.00–2.00)	0.00 (0.00–1.00)
MPLDG	2.00 (1.00–3.00)	2.00 (1.00–3.00)	2.00 (1.00–3.00)	2.00 (1.00–3.00)
Group comparisons	*p*-values			
HG vs. ATPG	1.000	1.000	1.000	1.000
HG vs. LZDG	<0.001	<0.001	<0.001	<0.001
HG vs. ATLDG	0.151	0.496	0.078	0.846
HG vs. MPLDG	<0.001	<0.001	<0.001	<0.001
ATPG vs. LZDG	<0.001	<0.001	<0.001	<0.001
ATPG vs. ATLDG	0.151	0.496	0.078	0.846
ATPG vs. MPLDG	<0.001	<0.001	<0.001	<0.001
LZDG vs. ATLDG	<0.001	<0.001	<0.001	<0.001
LZDG vs. MPLDG	0.087	1.000	0.933	1.000
ATLDG vs. MPLDG	<0.001	<0.001	<0.001	<0.001
K-W	156.089 ^a^	150.219 ^a^	150.433 ^a^	157.559 ^a^
df	4	4	4	4
Asymptotic Sig.	<0.001	<0.001	<0.001	<0.001

The results are expressed as median (minimum–maximum). Statistical analyses were performed using the Kruskal–Wallis (K-W) test followed by Dunn’s post hoc test with Bonferroni correction. ^a^ means the test statistic is adjusted for ties. For all groups *n* = 6. ATP: adenosine triphosphate; HG: healthy group; ATPG: ATP-only group; LZDG: linezolid-only group; ATLDG: ATP + linezolid group; MPLDG: methyl prednisolone + linezolid group; df: degree of freedom.

## Data Availability

The raw data supporting the conclusions of this article will be made available by the authors upon reasonable request.

## Abbreviations

The following abbreviations are used in this manuscript:

MDA	Malondialdehyde
tGSH	Total Glutathione
SOD	Superoxid
CAT	Catalase
ATP	Adenosine Triphosphate
TNF-α	Tumor Necrosis Factor-alpha
TP-I	Troponin I
ROS	Reactive Oxygen Species
ELISA	Enzyme-linked Immunosorbent Assay
LPO	Lipid Peroxidation
